# Supplementary material for: Causal Relationship between Aging and Anorexia Nervosa: A White-Matter-Microstructure-Mediated Mendelian Randomization Analysis
Source: Biomedicines. 2024 Aug 16;12(8):1874. doi: 10.3390/biomedicines12081874 (PMC11351342; doi:10.3390/biomedicines12081874)
Supplement: Supplementary file 1 [file biomedicines-12-01874-s001.zip › Supplementary Figure/Figure S8/Leaveoneout plot of MR analysis for TL to ubm-b-1497.pdf]

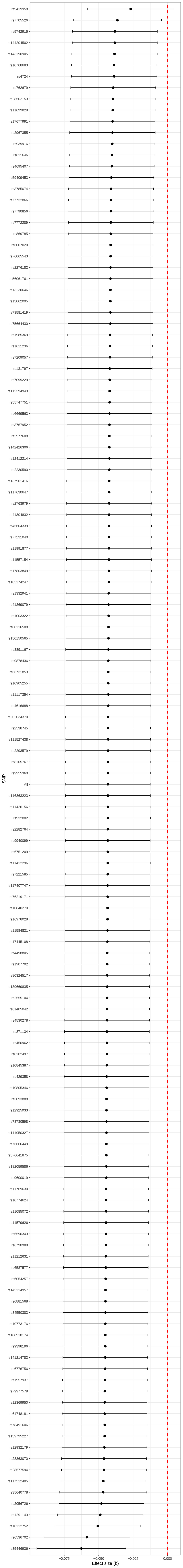
rs9419958
rs7705526
rs5742915
rs144204502
rs143190905
rs10768683
rs4724
rs762679
rs28502153
rs11699829
rs17677991
rs2967355
rs939916
rs611646
rs4695407
rs59409453
rs3785074
rs77732866
rs7790856
rs7772289
rs869785
rs6007020
rs76065543
rs2276182
rs56061761
rs13230646
rs13062095
rs73581419
rs75664430
rs1985369
rs1611236
rs7209057
rs131797
rs7099229
rs112394943
rs55747751
rs6669563
rs3767952
rs2977608
rs142426306
rs12412214
rs2230590
rs137901416
rs117630647
rs2763979
rs41304832
rs45604339
rs77231040
rs11991877
rs11557154
rs17803849
rs185174247
rs1332941
rs41269079
rs1003322
rs80116508
rs150150565
rs3891167
rs9878436
rs66731853
rs10905255
rs11117354
rs4616688
rs202034370
rs2538745
rs111527438
rs2293579
rs8105767
rs9955360
All
rs116863223
rs11426156
rs932002
rs2282764
rs9940099
rs6751209
rs11412296
rs7221585
rs117407747
rs76219171
rs10840270
rs16978028
rs11584821
rs17445108
rs4498805
rs1907702
rs80324517
rs139669835
rs2555104
rs61405042
rs4530278
rs871134
rs450962
rs8102497
rs10845387
rs429358
rs10805346
rs3093888
rs12925933
rs73730598
rs111950327
rs76666449
rs376641875
rs182059586
rs9600019
rs11769630
rs10774624
rs11085072
rs11579626
rs6590343
rs6790988
rs11212631
rs6587577
rs6054257
rs145114957
rs6881568
rs34550383
rs10773176
rs188918174
rs9398196
rs141214782
rs6776756
rs1957937
rs79977579
rs12369950
rs61748181
rs78491606
rs139795227
rs12932179
rs28363070
rs28577594
rs117512405
rs35640778
rs2056726
rs1291143
rs10112752
rs6536702
rs35446936
SNP
-0.075
-0.050
-0.025
0.000
Effect size (b)
